# The Interrelationship Between Water Use Efficiency and Radiation Use Efficiency Under Progressive Soil Drying in Maize

**DOI:** 10.3389/fpls.2021.794409

**Published:** 2021-12-10

**Authors:** Huailin Zhou, Guangsheng Zhou, Li Zhou, Xiaomin Lv, Yuhe Ji, Mengzi Zhou

**Affiliations:** ^1^State Key Laboratory of Severe Weather, Chinese Academy of Meteorological Sciences, Beijing, China; ^2^College of Applied Meteorology, Nanjing University of Information Science & Technology, Nanjing, China; ^3^Gucheng Agrometeorological Field Scientific Experiment Base, China Meteorological Administration, Baoding, China; ^4^Collaborative Innovation Center on Forecast Meteorological Disaster, Warning and Assessment, Nanjing University of Information Science & Technology, Nanjing, China

**Keywords:** fAPAR, leaf area index (LAI), maize, progressive soil drying, RUE, WUE

## Abstract

The maximizing of water use efficiency (WUE) and radiation use efficiency (RUE) is vital to improving crop production in dryland farming systems. However, the fundamental question as to the association of WUE with RUE and its underlying mechanism under limited-water availability remains contentious. Here, a two-year field trial for maize designed with five progressive soil drying regimes applied at two different growth stages (three-leaf stage and seven-leaf stage) was conducted during the 2013–2014 growing seasons. Both environmental variables and maize growth traits at the leaf and canopy levels were measured during the soil drying process. The results showed that leaf WUE increased with irrigation reduction at the early stage, while it decreased with irrigation reduction at the later stage. Leaf RUE thoroughly decreased with irrigation reduction during the progressive soil drying process. Aboveground biomass (AGB), leaf area index (LAI), a fraction of absorbed photosynthetically active radiation (fAPAR), and light extinction coefficient (k) of the maize canopy were significantly decreased by water deficits regardless of the growth stages when soil drying applied. The interrelationships between WUE and RUE were linear across the leaf and canopy scales under different soil drying patterns. Specifically, a positive linear relationship between WUE and RUE are unexpectedly found when soil drying was applied at the three-leaf stage, while it turned out to be negative when soil drying was applied at the seven-leaf stage. Moreover, the interaction between canopy WUE and RUE was more regulated by fAPAR than LAI under soil drying. Our findings suggest that more attention must be paid to fAPAR in evaluating the effect of drought on crops and may bring new insights into the interrelationships of water and radiation use processes in dryland agricultural ecosystems.

## Introduction

Maize (*Zea mays* L.), one of the world’s top three cultivated kinds of cereal along with wheat and rice (FAO, 2020), is typically cultivated in semi-arid and semi-humid areas in China. Even though maize is a C4 plant with higher temperature adaptation and lower water consumption than wheat and rice (Sanchez et al., 2014), its growth and productivity are more vulnerable to climate change than other staple crops (Tao et al., 2008). Therefore, it is not surprising that a worldwide significant decrease in maize yield trend can be attributed to the climate anomalies and extremes, especially seasonal drought and heat stress (Tao and Zhang, 2010; Lobell et al., 2011; Zampieri et al., 2019). North China Plain (NCP) is a drought-prone region, and also one of the foremost dryland agricultural production areas in China (Fang et al., 2021). Past and present climate trends and variability in NCP were characterized as increased air temperature and decreased precipitation (Ming et al., 2015). To make matters worse, the situation is expected to be further intensified under projected future climate, accompanied by a decrease in solar radiation (Liu et al., 2013; Huang et al., 2017). Adapting maize growth to the changing climate environments (decreasing solar radiation, decreasing precipitation, increasing temperature, etc.) will be an important innovation to increase total biomass and then grain yield (Yang et al., 2019; Kan et al., 2020). Therefore, it is particularly urgent and desirable to evaluate the relative potential of resource use efficiency and then enhance them for the sustainability of agricultural development in this drought-prone region.

Water use efficiency (WUE), defined as the ratio of carbon gain to water loss, is an important physiological indicator in assessing the interactions between the carbon and water cycles (Farquhar and Richards, 1984; Beer et al., 2009). Previous studies have enhanced the understanding that moderate drought has a stimulatory effect on crop WUE [e.g., durum wheat (Bhattarai et al., 2020), maize (Kang et al., 1998), and sweet sorghum (Dercas and Liakatas, 2006)]. However, the degree and duration of drought also play an important role in affecting WUE (Ma et al., 2019), as well as crop species and developmental stages. The underlying mechanism of progressive soil drying on WUE and its connection with other resource use efficiency remains unclear. Like soil water, solar radiation is the ultimate energy source for crop development and production (Monteith et al., 1977; Wild et al., 2005), but it also can cause stress to plants and modulate response to water deficit (Roeber et al., 2020). Radiation use efficiency (RUE), an important determinant of carbon sequestration by terrestrial ecosystems, indicates the efficiency of a plant to convert absorbed photosynthetically active radiation (PAR) of 400–700 nm wavelength into biomass (Monteith, 1972). In dryland agriculture, crop productivity is directly related to the efficiency to convert resources into biological materials, especially water and solar radiation. Therefore, improving our understanding of how to quantify the interaction of WUE and RUE and how they affect crop productivity is necessary for optimizing dryland agricultural management practice (Ding et al., 2021).

A large body of literature has emphasized the effects of water deficit only on WUE or RUE either at the leaf or canopy level (Steduto and Albrizio, 2005; Yi et al., 2010; VanLoocke et al., 2012; Marwein et al., 2016; Song et al., 2018; Hatfield and Dold, 2019; Cai et al., 2020; Yu et al., 2020). High WUE was proved to be related to low RUE under probable drought (Dercas and Liakatas, 2006). In contrast, WUE and RUE are directly proportional under optimal growth conditions (Ullah et al., 2019; Kukal and Irmak, 2020). However, little is known about the interrelationships of radiation and water use processes at both leaf and canopy levels in agricultural ecosystems. In addition, the connection between WUE and RUE response to progressive soil drying at different growth stages remains unclear. WUE and RUE are inversely related to canopy conductance (gc) (Sadras et al., 1991; Hernández et al., 2021), which may be suitable to distinguish and communicate the WUE and RUE relations (Kukal and Irmak, 2020). In addition, changes in canopy structure [e.g., leaf angle, plant height, leaf area index (LAI)] reflect both in light transmittance, and reflectance under water stress conditions (Holmes and Keiller, 2002; Onoda et al., 2014). The variation of crop evapotranspiration is greatly affected by environmental and vegetation factors, which are directly or indirectly mediated by changes in biotic factors, such as LAI (Zhou et al., 2019). LAI is a primary descriptor of vegetation function and structure, and is functionally related to the exchange of water and energy between vegetation and the atmosphere (Running, 1984; Liu et al., 2016). What’s more, the fraction of absorbed photosynthetically active radiation (fAPAR) by the canopy is a key variable not only in assessing the production of the vegetation, but also in the efficiency of light usage (Gitelson et al., 2014). However, it’s still not very clear about how the interrelationship between WUE and RUE are mediated by LAI and fAPAR and their relative influence pathway.

Here, we used an experiment-based approach to explore the link between WUE and RUE and its underlying mechanism under soil drying at different developmental stages. Specifically, the objectives were to address the following questions: (i) How does WUE and RUE respond to soil drying? (ii) How does WUE commutate with RUE at both leaf and canopy scales? (iii) Does the association between WUE and RUE change with different progressive soil drying patterns and growth stages? (iv) What are the direct and indirect pathways by which LAI and fAPAR mediate the association of WUE with RUE under limited-water availability?

## Materials and Methods

### Experiment Site

**FIGURE 1:**
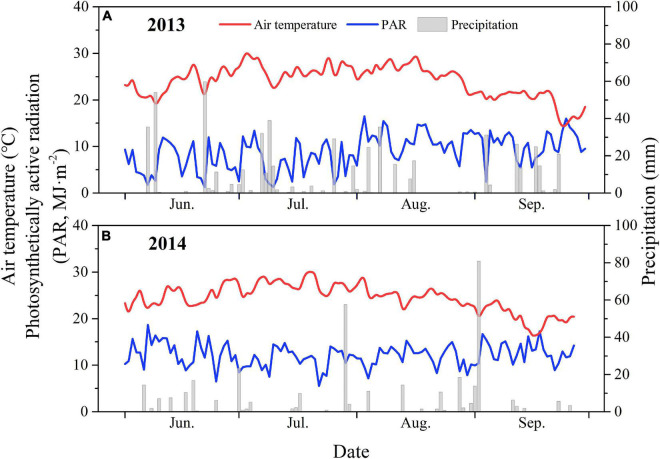
Daily mean air temperature (*T*, °C), precipitation (*P*, mm), photosynthetically active radiation (PAR, MJ m^–2^) at the study site in 2013 **(A)** and 2014 **(B)** growing seasons.

The field experiment was conducted in two growing seasons (from June to October 2013 and 2014) at the Gucheng Agro-meteorological Field Scientific Experiment Base (39°08′N, 115°40′E and 15.2 m a.s.l.) in Baoding, Hebei Province, China. The site is a typical maize production region located on NCP, with a warm temperate continental monsoon climate. The mean annual temperature (1981–2010), annual precipitation, and average sunshine duration are 12.2°C, 515.5 mm, and 2264 h yr^–1^, respectively. The variations in the daily mean air temperature (*T*), *PAR*, and precipitation (*P*) for the growing seasons in 2013 and 2014 are shown in Figure 1. The soil type is classified as sandy loam, with total potassium, total phosphorus, and total nitrogen contents of 17.26 g kg^–1^, 1.02 g kg^–1^, and 0.98 g kg^–1^, respectively (Fang et al., 2013). The mean pH and soil bulk density within a depth of 50 cm are 8.19 and 1.37 g cm^–3^ (Wang Q. et al., 2018). The average field capacity is 22.7% (g g^–1^) and the wilting point is 5.0% (g g^–1^). Double cropping with two crop harvestings (wheat–maize rotation) in a year is conventional farming practice in this region. The principal crops are winter wheat (*Triticum aestivum*), maize (*Zea mays*), potato (*Solanum tuberosum*), peanut (*Arachis hypogaea*), and soybean (*Glycine max*).

### Experimental Design and Farm Management

The study was carried out in the field with a randomized complete block design. The area of each plot was 8 m^2^ (4 m long, 2 m wide). To block out the natural precipitation, a large electric-powered waterproof shelter was used to cover the plots when it was rainy, otherwise, it was moved away to keep the experimental plots fully exposed to the ambient conditions. In addition, cement walls with a depth of 3.0 m were installed between each plot to avoid horizontal water exchange across plots. The selected cultivar was *zhengdan-958*, which is a drought-tolerant variety that has been widely planted on the NCP over the last two decades. Approximately 1 month before seeding, the soil moisture of each plot from 0 to 100 cm at 10 cm interval was measured and calculated, and then each plot was irrigated to reach the same water condition. A controlled-release fertilizer called diammonium phosphate (DAP), with N and P_2_O_5_ accounting for 16 and 45% of the total mass, was conventionally applied before sowing at a rate of 320 kg⋅hm^–2^. Maize seeds were sown on June 27, 2013, and June 24, 2014, during the two growing seasons. The plant density was set to 52 plants per plot (65000 plants hm^–2^). Prior to the imposed prolonged drought, all plots were well irrigated to encourage seedling emergence. The irrigation amounts were designed based on the mean precipitation (150 mm) in July from 1981 to 2010. In 2013, five irrigation amounts were conducted at the beginning of the seven-leaf stage (July 24, 27 days after sowing (DAS)), 80 mm (T1, equal to 53.3% of 150 mm precipitation in July), 60 mm (T2, 40%), 40 mm (T3, 26.7%), 25 mm (T4, 16.7%), and 15 mm (T5, 10%). In 2014, another five irrigation treatments were carried out at the beginning of the three-leaf stage (July 2, 8 DAS), 150 mm (W1, 100%), 120 mm (W2, 80%), 90 mm (W3, 60%), 60 mm (W4, 40%), and 30 mm (W5, 20%) (Zhou et al., 2021). Each treatment was designed with three replicate plots. All irrigations were recorded by a water meter. After the irrigation, no more irrigation water or precipitation was applied during the remaining growing seasons. The maize crops were harvested on October 8, 2013, and October 9, 2014, respectively.

### Measurements of Environmental Variables and Maize Growth Traits

#### Available Soil Water Content and Soil Water Storage

During the two growing seasons, the soil water content was measured using the oven-drying method with an interval of 7–14 days. As more than 95% root biomass of maize was expected to grow within the 0–30 cm soil layers (Wang S. et al., 2018), and it was laborious to take soil samples under drought conditions, the sampling depth was set up to 50 cm. Soil samples were collected at a 10-cm interval, and soil cores were randomly chosen from the middle area of two rows of maize plants in the center of each plot. The gravimetric water content of each soil layer was measured by taking weights before and after drying the soil at 105°C to a constant weight. The average soil water content was determined by the mean value of three different sampling positions for each treatment. There were 8 measurements performed throughout the 2013 and 2014 growing seasons. The available soil water content (ASWC, %) of each layer and soil water storage (SWS, mm) was calculated according to the following equations (Haghverdi et al., 2015; Cosentino et al., 2016):


(1) S⁢W⁢C=(S⁢F⁢M-S⁢D⁢M)/S⁢D⁢M

(2) A⁢S⁢W⁢C=(S⁢W⁢C-W⁢P)/(F⁢C-W⁢P)×100%

(3) $$S\,W\,S=\sum_{i}^{n}10\times h_{i}\times \rho_{i}\times S\,W\,C_{i}$$

where *SWC* (g g^–1^) is the soil water content on a mass basis; *SFM* (g) and *SDM* (g) are the soil fresh and dry mass, respectively. *FC* (g g^–1^) is the field capacity, and *WP* (g g^–1^) is the wilting point. *SWC*_*i*_ is the soil water content of the *i*th soil layer; *h*_*i*_ (cm) is the thickness of the *i*th soil layer; *ρ_*i*_* (g cm^–3^) is the soil bulk density of the *i*th soil layer, and n is the number of soil layers measured.

Evapotranspiration (*ET*, mm) which includes soil water evaporation and crop transpiration for different growth periods of maize was calculated based on the water balance equation as follows:


(4) E⁢T=I+P+K-D-R+Δ⁢S⁢W⁢S

where *I* (mm) is the irrigation amount, *P* (mm) is precipitation, which is blocked out by the shelter since sowing (here *P* = 0), *K* (mm) is the groundwater influx into the root zone, *D* (mm) is the drainage, and *R* (mm) is surface runoff. In this experiment, *K*, *D*, and *R* were negligible. Therefore, the soil–water balance equation is reduced as (Guan et al., 2015):


(5) E⁢T=I+Δ⁢S⁢W⁢S

(6) $$\Delta \,S\,W\,S=S\,W\,S_{s\,t\,a\,r\,t}-S\,W\,S_{e\,n\,d}$$

where *SWS*_*start*_ (mm) and *SWS*_*end*_ (mm) are SWS at the start and end of the growth period at interest, respectively.

#### Measurement of Leaf Gas Exchange Parameters

Leaf gas exchange parameters were measured by using a portable photosynthesis system (Li-6400XT; Li-Cor, Lincoln, NE, United States) with a fluorescence leaf chamber (Li-6400-40). Three to five healthy and representative maize plants (at least 0.5 m from the plot edge) were selected in each treatment with the same sampling interval as the soil water content measurement. In detail, the measurements were conducted on 33 DAS and 16 DAS in 2013 and 2014 growing seasons, respectively. Therefore, there were 6 and 7 measurements during experimental periods in 2013 and 2014, respectively. Measurements were conducted in the middle area of the newly fully expanded leaves in the morning (9:30–11:30) on clear days. The environmental conditions in the leaf chamber were controlled with a reference CO_2_ concentration of 380–410 μmol⋅mol^–1^ and an airflow rate of 500 μmol⋅s^–1^. The air temperature, relative air humidity, and photosynthetic photon flux density were maintained under ambient conditions. The net photosynthetic rate (*P*_*n*_), transpiration rate (*T*_*r*_), and leaf photosynthetically active radiation (*PAR*_*leaf*_) were recorded. Water use efficiency (WUE_leaf_) and radiation use efficiency (RUE_leaf_) at the leaf level was determined by the following equations (Farquhar and Richards, 1984; Yang et al., 2018):


(7) $$W\,U\,E_{l\,e\,a\,f}=P_{n}/T_{r}$$

(8) $$R\,U\,E_{l\,e\,a\,f}=P_{n}/P\,A\,R_{l\,e\,a\,f}$$

#### Leaf Area Index and Aboveground Biomass

Three healthy maize plants were randomly selected from each treatment and destructively harvested with the same sampling interval as leaf gas exchange measurement. The aboveground plant organs were clearly separated and quickly weighed to avoid excessive water loss. Aboveground biomass (AGB) included all the leaves, leaf sheath, shoot, tassel, and ear. The plant leaf area was determined by the Montgomery method (Stewart and Dwyer, 1999), with the maximum length (*L*, cm) and width (*W*, cm) of each leaf measured by a ruler. All fresh plant organs were oven dried at a temperature of 105°C for 1 h and were kept at 80°C until a constant dry mass was obtained (Sun et al., 2019). The leaf area (LA, cm^2^) of each maize plant, LAI (m^2^ m^–2^), and AGB (g m^–2^) at the canopy level were calculated as follows:


(9) $$L\,A=\sum_{i}^{n}\left(0.75\times L_{i}\times W_{i}\right)$$

(10) $$L\,A\,I=\sum_{j}^{m}\left(L\,A_{j}\right)/m\times d/10000$$

(11) $$A\,G\,B=\sum_{j}^{m}A\,G\,B_{j}\times d/m$$

where *n* is the total number of leaves per maize plant, *m* is the number of repetitions, *d* is the plant density (plants m^–2^), and AGB*_*j*_* is the total aboveground biomass of the selected plant (g plant^–1^).

#### Measurement of Photosynthetically Active Radiation, Fraction of Absorbed Photosynthetically Active Radiation, and the Cumulative Amount PAR Intercepted by the Canopy

Photosynthetically active radiation was measured by a line quantum sensor with 64 photodiodes (SunScan, Delta T Devices Ltd., Cambridge, United Kingdom) at 11:30–14:00 on clear days. The measured solar radiation included the incoming PAR above the canopy (*PAR*_*in*_), PAR reflected by the canopy and soil (*PAR*_*out*_), PAR transmitted through the canopy (*PAR*_*tran*_), and PAR reflected by the soil (*PAR*_*soil*_). *PAR*_*in*_ and *PAR*_*out*_ were measured using the quantum sensor placed horizontally 1.0 m above the maize canopy surface pointing toward the sky and ground, respectively; *PAR*_*tran*_ was measured with line quantum sensors placed at about 5 cm above the ground, pointing upward; and *PAR*_*soil*_ was measured with line quantum sensors placed about 12 cm above the ground, pointing downward (Gitelson et al., 2014). Each kind of PAR was obtained with the mean value of three different measurement directions. The fAPAR was determined by the following formula, which excluded the noise of the bare soil background (Gallo and Daughtry, 1984):


(12) $$f\,A\,P\,A\,R=\left(\left(P\,A\,R_{i\,n}-P\,A\,R_{o\,u\,t}\right)-\left(P\,A\,R_{t\,r\,a\,n}-P\,A\,R_{s\,o\,i\,l}\right)\right)/P\,A\,R_{i\,n}$$

Then, daily LAI values were calculated by a cubic spline interpolation between measured points of LAI, assuming a linear relationship between subsequent sampling dates (Cosentino et al., 2016). Daily intercepted PAR was calculated using Beer’s law, with a simple assumption of constant *k* estimated by Eq. 13 throughout the growing season (Ceotto and Castelli, 2002). As the remaining radiation flux after passing through a leaf area declines exponentially, which can be expressed by the Lambert-Beer model (Monsi and Saeki, 2005; Cosentino et al., 2016).


(13) f⁢A⁢P⁢A⁢R=1-e⁢x⁢p⁢(-k×L⁢A⁢I)

where *k* is the extinction coefficient.

The cumulative amount of PAR intercepted by the maize canopy (IPAR) during the growth period was calculated as follows (Yang et al., 2004):


(14) $$I\,P\,A\,R=\sum_{i=1}^{n}0.5\times R_{n}\times f\,A\,P\,A\,R_{i}$$

where 0.5 is the fraction of PAR relative to total incident solar radiation (*R*_*n*_), and fAPAR*_*i*_* is the daily fAPAR of the *i*th day.

#### Measurement of Meteorological Variables

During the 2013–2014 growing seasons, the main meteorological parameters, such as the mean daily air temperature (*T*, °C), daily PAR, and daily precipitation (*P*, mm) were measured by a weather station located nearly 20 m from the experimental field.

## Statistics

Canopy water use efficiency (WUE_AGB_, g m^–2^ mm^–1^) and radiation use efficiency (RUE_AGB_, g MJ^–1^) were calculated as the slope of the linear regression between AGB and cumulative ET and IPAR, respectively, during the experimental period. Canopy conductance (*g*_*c*_) is defined as the ratio of RUE_AGB_ to WUE_AGB_ (Sadras et al., 1991). The effects of the progressive soil drying treatments on WUE_leaf_, RUE_leaf_, fAPAR, LAI, and AGB were evaluated by repeated measures analysis of variance (RM-ANOVA). The least significant difference (LSD) test was applied to examine differences between treatments according to Duncan’s test. The relationship between WUE and RUE was evaluated by simple linear regression at both leaf and canopy levels. The standardized major axis (SMA) was employed to determine the WUE and RUE relationships among different treatments by using the “smatr” package in R 4.0.2 (R Core Team). The structural equation model (SEM) was employed using AMOS 21.0 (Amos Development Co., Greene, ME, United States) to analyze the direct and indirect pathway by that LAI and fAPAR influence AGB then WUE and RUE under progressive soil drying. In all cases, differences were deemed to be significant if *p* < 0.05. All the figures were plotted in Origin 9.1 (Origin Lab Corporation, Northampton, MA, United States).

## Results

### Soil Water Storage Dynamic Variations Under Various Progressive Soil Drying Treatments

**FIGURE 2:**
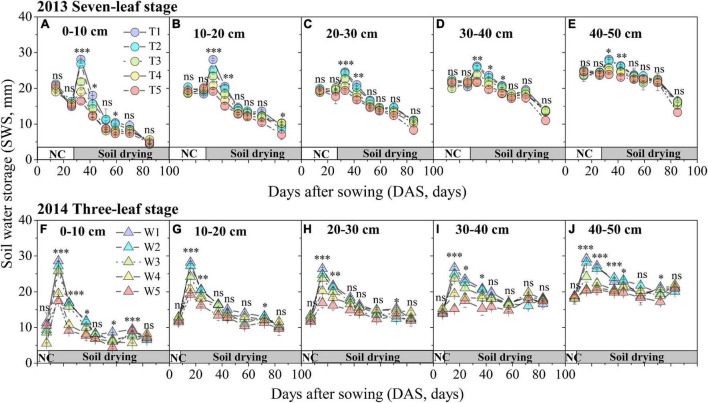
Time series of SWS in the different soil layers under different progressive soil drying treatments during 2013 **(A–E)** and 2014 **(F–J)** growing seasons. The growth period prior to treatment is indicated by the horizontal white bar named “NC,” while the period during treatment is represented by the horizontal gray bar named “progressive soil drying”. “^***^”, “^**^”, and “*” denote significant differences between treatments at the 0.001, 0.01, and 0.05 levels, respectively, while “ns” indicates no significant differences. The error bars indicate the standard errors of the replications.

Soil water storage in different soil layers decreased significantly and exhibited a similar trend with DAS in two growing seasons (Figure 2). In 2013, prior to the treatment period (16–27 DAS), the SWS values of five soil layers showed no significant difference among treatments (T1–T5) based on SM-ANOVA (Figures 2A–E). The SWS values in each soil layer showed significant differences among the T1–T5 treatments after treatments began until 42 DAS. Since then, no obvious differences were observed between the T1 and T5 treatments at different soil layers, except for 0–10 cm in 59 DAS and 20–30 cm in 85 DAS. Moreover, SWS in the upper soil layers (0–10, 10–20, and 20–30 cm) showed even more significant differences than those in the lower layers (30–40, 40–50 cm). In 2014, since irrigation treatments were applied (8 DAS), the average duration of significant differences in the SWSs of the W1–W5 treatments for different soil layers were longer (approximately 1 month) than those of the T1–T5 treatments in 2013 (Figures 2F–J).

### Water Use Efficiency and Radiation Use Efficiency Response to Progressive Soil Drying at the Leaf Level

**FIGURE 3:**
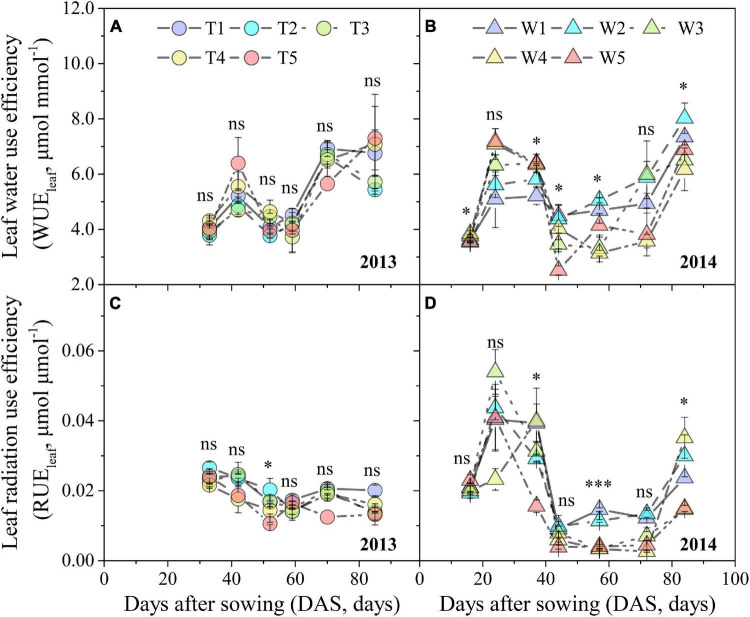
Changes in WUE_leaf_ and RUE_leaf_ under different progressive soil drying treatments during the 2013 **(A,C)** and 2014 **(B,D)** growing seasons. “^***^”, “^**^”, and “*” denote significant differences between treatments at the 0.001, 0.01, and 0.05 levels, respectively, while “ns” indicates no significant differences. Error bars denote the standard errors of the replications.

**FIGURE 4:**
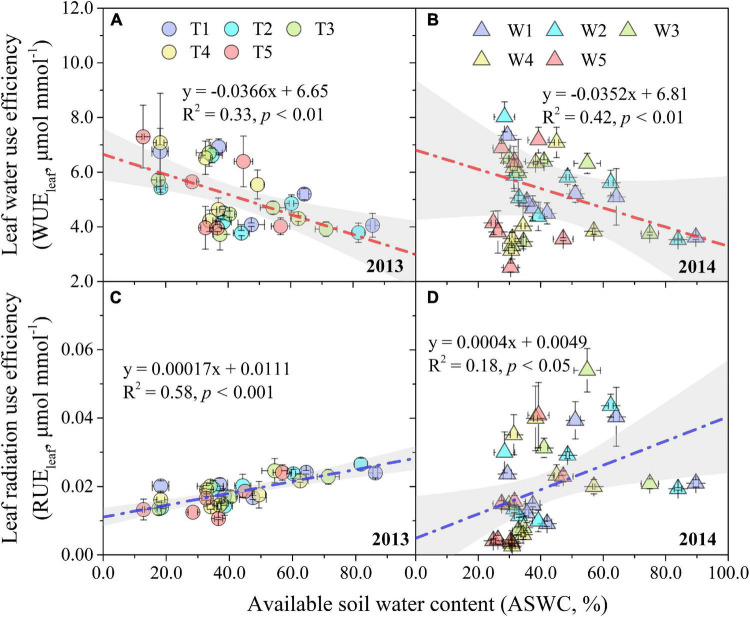
Response of WUE_leaf_ and RUE_leaf_ to ASWC under different progressive soil drying treatments in 2013 **(A,C)** and 2014 **(B,D)**. The gray areas are the 95% confidence bands of the linear fitting. Error bars denote the standard errors of the replications.

Leaf water use efficiency and RUE_leaf_ under different progressive soil drying treatments displayed a similar trend of first increasing, then gradually decreasing, and finally increasing again (Figures 3A,B,D), which was not obvious for RUE_leaf_ in the T1–T5 treatments (Figure 3C). At the early stage, progressive soil drying stress increased WUE_leaf_, but depressed it at the later stage (Figures 3A,B). However, progressive soil drying stress had a negative effect on RUE_leaf_ throughout the experimental period (Figures 3C,D). Even though progressive soil drying treatments began at different growth stages, the sensitivity of the WUE_leaf_ response to ASWC in 2013 (slope = –0.037) was not significantly different for that in 2014 (slope = –0.035), while the sensitivity of RUE_leaf_ to ASWC showed an obvious difference between 2013 (slope = 0.00017) and 2014 (slope = 0.00040) growing seasons (Figure 4).

### Aboveground Biomass, Leaf Area Index, and Fraction of Absorbed Photosynthetically Active Radiation Response to Progressive Soil Drying

**FIGURE 5:**
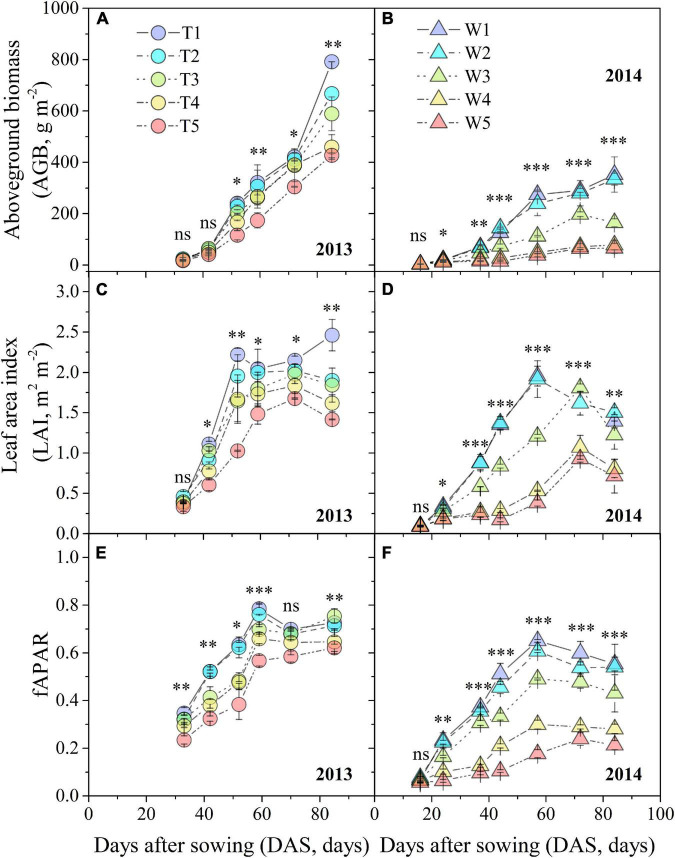
Changes in AGB, fAPAR, and LAI under different progressive soil drying treatments during the 2013 **(A,C,E)** and 2014 **(B,D,F)** growing seasons. “^***^”, “^**^”, and “*” denote significant differences between treatments at the 0.001, 0.01, and 0.05 levels, respectively, while “ns” indicates no significant differences. The error bars indicate the standard errors of the replications.

Progressive soil drying significantly decreased AGB, LAI, and fAPAR based on RM-ANOVA (Figure 5). In 2013, water deficit had a significant negative effect on biomass accumulation (Figure 5A). The dynamic characteristics of LAI and fAPAR shared a similar trend (Figures 5C,E). LAI values of T2–T5 treatments reached maximum values approximately 18 days later than fAPAR, except for T1. In 2014, the rates of increment in AGB among the W1–W5 treatments were much slower than those of the T1–T5 treatments, especially after 59 DAS (Figure 5B). There were obvious maximum values for LAI and fAPAR at 59 DAS, followed by a subsequent declining trend with progressive soil drying (Figures 5D,F).

**FIGURE 6:**
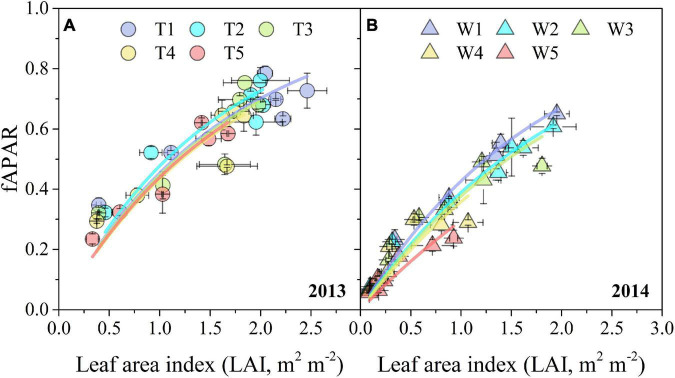
Relationships between periodic measurements of LAI and fAPAR under different progressive soil drying treatments during the 2013 **(A)** and 2014 **(B)** growing seasons. The colorful fitting line was formed as Eq. 13. The error bars indicate the standard errors of the replications.

The relationships between fAPAR and LAI under different irrigation treatments were well represented by a non-linear Eq. 13 (Figure 6). Progressive soil drying had significant adverse effects on *k* derived from the asymptotic equation, especially in 2014 (Table 1). Moreover, the *k* values under different progressive soil drying treatments generally decreased with the irrigation reductions.

**TABLE 1:** Estimated *k* under different progressive soil drying treatments during the 2013 (T1–T5 treatments) and 2014 (W1–W5 treatments) growing seasons.

Year	Parameter	Progressive soil drying treatment				
2013		T1	T2	T3	T4	T5
	*k*	0.61 ± 0.07	0.65 ± 0.05	0.59 ± 0.07	0.57 ± 0.06	0.58 ± 0.04
	*R* ^2^	0.70	0.82	0.72	0.74	0.89
2014		W1	W2	W3	W4	W5
	*k*	0.56 ± 0.02	0.49 ± 0.03	0.47 ± 0.04	0.44 ± 0.06	0.35 ± 0.04
	*R* ^2^	0.98	0.96	0.87	0.56	0.77

**FIGURE 7:**
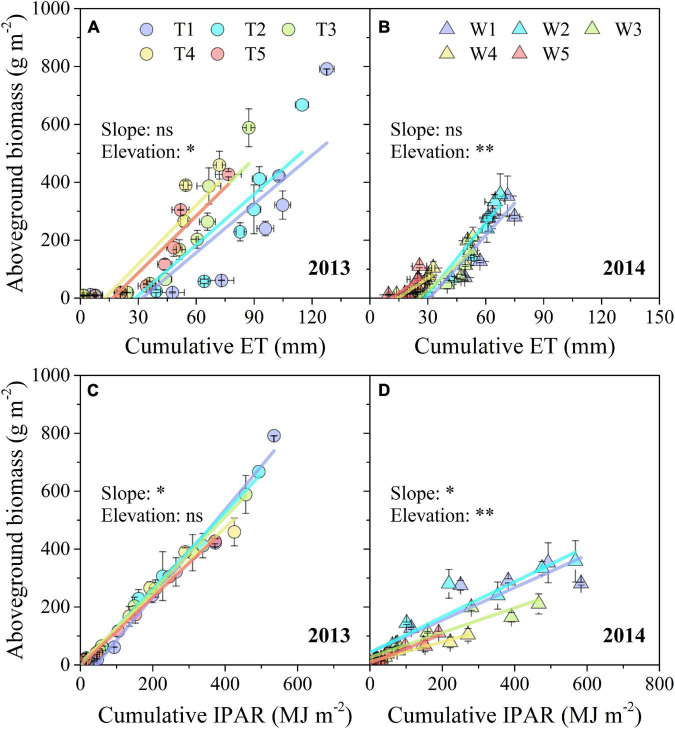
Response of AGB to cumulative ET and IPAR under different progressive soil drying treatments during the 2013 **(A,C)** and 2014 **(B,D)** growing seasons. “^**^” and “*” denote significant differences between treatments at the 0.01 and 0.05 levels, respectively, while “ns” indicates no significant differences. The error bars indicate the standard errors of the replications.

As indicated by SMA analysis, the estimated SMA slopes of the linear relationship between AGB and cumulative ET remained stable in both 2013 and 2014, while the SMA elevations were significantly affected by progressive soil drying (Figures 7A,B). In contrast, progressive soil drying significantly affected the estimated SMA slope of the linear relationship between AGB and cumulative IPAR in both 2 years, and significantly affected the elevation only in 2014 (Figures 7C,D).

### The Association of Water Use Efficiency With Radiation Use Efficiency Across the Leaf and Canopy Levels

**FIGURE 8:**
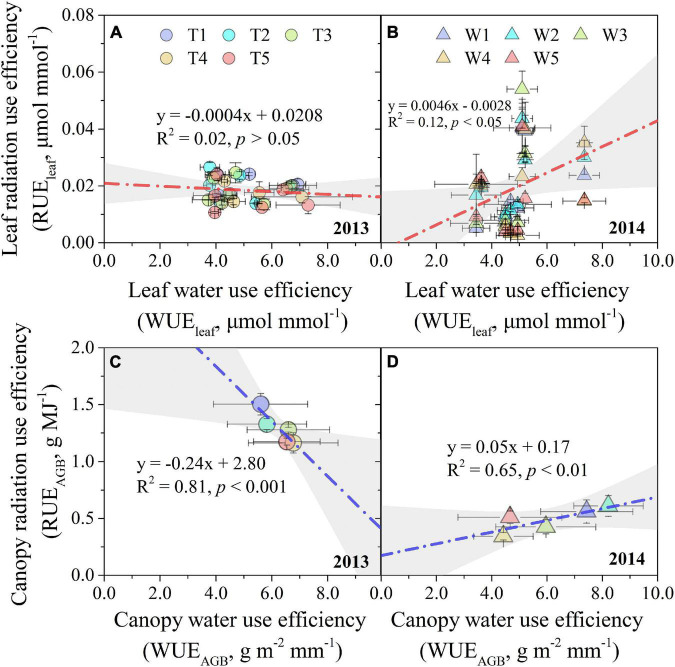
Relationships between WUE and RUE at leaf and canopy levels in 2013 **(A,C)** and 2014 **(B,D)** growing seasons. The gray areas are the 95% confidence bands of the linear fitting. The error bars denote the standard errors of the replications.

The linear relationships between WUE and RUE were opposite in 2013 compared with those in 2014, even if not statistically significant in 2013 (*p* > 0.05) (Figure 8). In detail, the slope (RUE_leaf_ vs. WUE_leaf_) was negative for the pooled data of the T1–T5 treatments in 2013, while it was positive for the W1–W5 treatments in 2014 (Figures 8A,B). Moreover, the relationship between WUE_AGB_ and RUE_AGB_ was significantly negative (*R*^2^ = 0.81, *p* < 0.01) in 2013, while it was significantly positive (*R*^2^ = 0.65, *p* < 0.01) in 2014 (Figures 8C,D). In addition, the absolute value of the linear slope (RUE_AGB_ vs. WUE_AGB_) in 2013 was nearly 3.8 times larger than that in 2014.

### Effects of Leaf Area Index and Fraction of Absorbed Photosynthetically Active Radiation on the Association Between Water Use Efficiency and Radiation Use Efficiency

**FIGURE 9:**
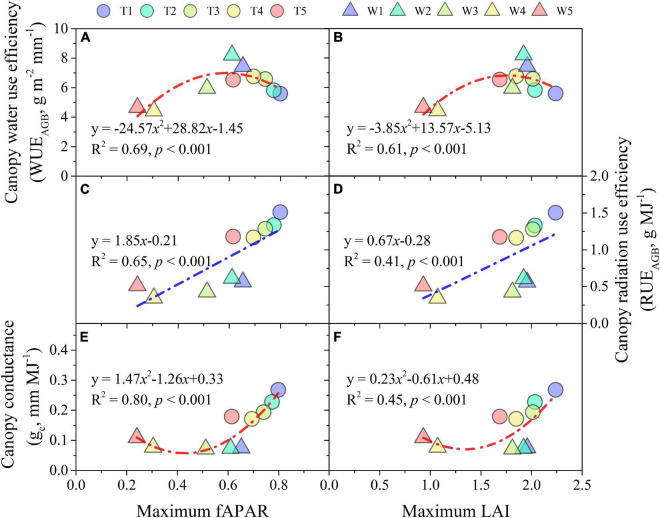
Water use efficency **(A,B)**, radiation use efficiency **(C,D)** of AGB, and canopy conductance **(E,F)** response to maximum LAI and fAPAR during 2013 and 2014 growing seasons.

Water use efficiency of AGB shared a quadratic relationship with maximum fAPAR and LAI (Figures 9A,B), while it was a linear relationship for RUE_AGB_ (Figures 9C,D). Moreover, WUE_AGB_ and RUE_AGB_ were more linked with fAPAR than LAI as the *R*^2^ explained. Maize seasonal *g*_*c*_ ranged between 0.07 and 0.27 mm MJ^–1^. A quadratic relationship was also fitted between *g*_*c*_ and maximum fAPAR (*R*^2^ = 0.80, *p* < 0.001) and LAI (*R*^2^ = 0.45, *p* < 0.01) across irrigation treatments, respectively; where *g*_*c*_ were more strongly linked with maximum fAPAR relative to maximum LAI (Figures 9E,F).

**FIGURE 10:**
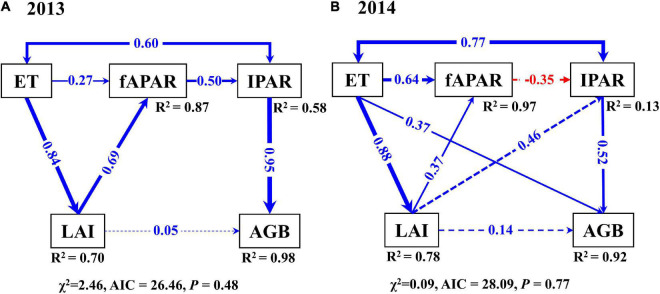
Structural equation models for the direct and indirect pathway of ET, fAPAR, LAI, and IPAR on AGB in 2013 **(A)** and 2014 **(B)**. Numbers on arrows are standardized path coefficients, blue arrows are positive and red are negative, solid arrows indicate significant standardized path coefficients (*p* < 0.05) (significance levels are *p < 0.05, **p < 0.01, and ***p < 0.001), dashed arrows indicate non-significant standardized path coefficients (*p* > 0.05). *R*^2^ values close to the rectangles indicate the variance explained by the model. ET, actual evapotranspiration; fAPAR, the fraction of absorbed photosynthetically active radiation; IPAR, intercepted photosynthetically active radiation; LAI, leaf area index; AGB, aboveground biomass.

Moreover, SEM analysis showed that the ET, fAPAR, IPAR, and LAI explained 98% and 92% of the variation of AGB in 2013 and 2014, respectively (Figure 10). In 2013, the primary latent variable that affected AGB was IPAR (total standardized path coefficient: 0.95), whereas it was ET (0.37) and IPAR (0.52) in 2014. Although fAPAR did not directly affect AGB, LAI only had a weak direct effect on AGB in both growing seasons. ET affected AGB *via* fAPAR with a path coefficient of 0.40 and −0.18 in 2013 and 2014, respectively, while they were 0.04 and 0.06 through LAI. Besides, the effect of LAI on AGB through fAPAR was 0.33, which was larger than that the direct effect of LAI on AGB (0.05) in 2013 (Figure 10A). Overall, the models showed that the influences of ET and IPAR on AGB were more mediated by fAPAR than LAI under progressive soil drying.

## Discussion

### Effects of Progressive Soil Drying on the Dynamic Variations of Water Use Efficiency and Radiation Use Efficiency

Understanding the dynamic variations of WUE and RUE and their underlying mechanisms can improve our ability to predict the effects of climate change on carbon and water cycles. In fact, the temporal variations in WUE and RUE are affected by various climatic factors (Yu et al., 2008) and genetic variables (Furbank et al., 2019), including common environmental stresses (e.g., drought, heat, salt, and solar dimming) and physiological, developmental, and phenological variations in plants (Turner et al., 2003; Leakey et al., 2019). Furthermore, previous studies revealed that the seasonal variation in WUE_leaf_ was closely coupled with leaf stoichiometry (Du et al., 2020), and also driven by air temperature, vapor pressure deficit (*VPD*), and solar radiation (Jiang et al., 2020). Our results indicated that progressive water deficit slightly changed the seasonal variations of WUE_leaf_, which were characterized by obvious fluctuations (Figures 3A,B). This finding was similar to a study for grape (Medrano et al., 2015). At the canopy level, diurnal WUE peaked in the early morning, while RUE topped at sunset, seasonal WUE and RUE reached a maximum value in summer (Gao et al., 2018). However, our results suggested that the dynamic variations of RUE_leaf_ showed different changing patterns in the 2013 and 2014 growing seasons (Figures 3C,D). In 2013, RUE_leaf_ decreased with DAS, whereas the RUE_leaf_ had significant fluctuations in 2014, which might be contributed to the differences in the degree and duration of soil drying. The values of RUE for maize in the later growth stage were often lower than those of the early growth stage (Otegui et al., 1995), which were also found in the 2013 growing season (Figure 3C), as the progressive loss of leaf photosynthetic capacity with soil drying and increasing leaf age (Earl and Tollenaar, 1999).

### Effects of Progressive Soil Drying on Aboveground Biomass, Leaf Area Index, and Fraction of Absorbed Photosynthetically Active Radiation

Drought stress significantly reduces crop production through its adverse effects on root water absorption, nutrient uptake, leaf net assimilation, and subsequently on dry matter accumulation (like AGB) and distribution (Ullah et al., 2019). The light interception capacity of a crop is mainly determined by the LAI and fAPAR (Hikosaka et al., 2016). Smaller leaf area and biomass lead to a smaller LAI and fAPAR, which is not beneficial for light interception (Baret et al., 2013; Mantilla-Perez and Salas Fernandez, 2017). In addition, leaf rolling is a mechanism developed by plants to mitigate the impact of environmental stresses, especially under severe stress conditions (Baret et al., 2018). Our study indicated that progressive soil drying significantly decreased AGB, LAI, and fAPAR (Figure 5), and accelerated leaf senescence (Figures 5E,F). Moreover, the adverse effects of progressive soil drying applied at the three-leaf stage on *k* were more significant than those at the seven-leaf stage (Figure 6 and Table 1). In general, WUE_AGB_ increased with irrigation reduction (T1–T5 treatments) in 2013 when soil drying began at the seven-leaf stage, whereas WUE_AGB_ decreased with irrigation reduction (W1–W5 treatments) in 2014 when soil drying began at the three-leaf stage (Figures 7A,B). The results suggest that moderate water stress increased WUE_AGB_, while severe water stress decreased WUE_AGB_. The main reason is that drought affected leaf development and expansion as soil drying. Consequently, the RUE values of different irrigation treatments were significantly reduced by water deficits (Figures 7C,D). The theoretical RUE of maize under optimal conditions is about 3.84 ± 0.08 g MJ^–1^ (Lindquist et al., 2005), which is much higher than our results which were severely decreased by soil drying. Recent breeding efforts have no evident effect on crop water use but have significant improvements on crop biomass production and partitioning (Curin et al., 2020). The maize ideotypes planted under the future climate of the NCP should have a longer reproductive growing period, faster potential grain filling rate, higher maximum grain numbers, and higher RUE (Xiao et al., 2020). Therefore, there is still a great potential to breed new maize varieties with both high WUE and RUE and improve field management for future climate.

### Influencing Factors on the Interaction Between Water Use Efficiency and Radiation Use Efficiency

The relationship between WUE and RUE may exhibit various patterns at different spatial-temporal scales and environmental backgrounds (Liu et al., 2019). At the leaf level, low transpiration induced by stomatal closure leads to increased WUE as water loss exceeds photosynthesis (Kang et al., 1998). Low photosynthesis tends to decrease RUE, causing WUE to be negatively correlated with RUE (Tarvainen et al., 2015). Our results also confirmed that leaf WUE and RUE were negatively correlated when progressive soil drying was applied at the seven-leaf stage (Figure 8A). However, when photosynthetic downregulation was caused by non-stomatal limitation during serious water stress (Song et al., 2020), leaf WUE dropped as photosynthesis decreased more than transpiration. As a result, the relationship between leaf WUE and RUE was positively correlated (Figure 8B). However, the critical value of the soil or plant water content where arouses the relationship between WUE and RUE to shift remains unknown and needs to be quantified in future study. The similar relationships between WUE and RUE were also found at canopy level, and even stronger than those at leaf level (Figures 8C,D). At the canopy level, high WUE of sweet sorghum tends to be related to low RUE under probable drought in sweet sorghum (a C4 plant similar to maize) (Dercas and Liakatas, 2006), which was also confirmed in our study (Figure 8C). A trade-off may occur between the use efficiency of two resources when there are differences in the relative costs of the resources, with an increase in the use efficiency of an “expensive” resource and a decrease in a “cheaper” resource (Bloom et al., 1985). Soil water is an “expensive” resource, while radiation is a “cheaper” resource under water-deficit conditions. The trade-off was confirmed in the interrelationship between canopy WUE and RUE when progressive soil drying treatments were applied at the seven-leaf stage (Figures 8A,C). However, the relationship between WUE and RUE turned out to be synergistic when progressive soil drying treatments were applied at the three-leaf stage (Figures 8B,D).

Leaf area index plays an important mediating role in the relationship among climate, soil variables, and evapotranspiration (Zhou et al., 2019). Meanwhile, fAPAR is a key variable not only in assessing vegetation productivity but also in light use efficiency (Gitelson et al., 2014). Accurately quantifying LAI and fAPAR is important for characterizing the dynamics of carbon and energy exchanges between vegetation and the atmosphere (Serbin et al., 2013). Our results indicated that fAPAR was more related to canopy conductance (*g*_*c*_, representing transpired water per unit of IPAR) than LAI (Figure 9). Even though LAI and fAPAR are deemed to be closely related (Zhou et al., 2002; Monsi and Saeki, 2005), their direct and indirect effects on AGB were significantly different according to an SEM analysis (Figure 10). fAPAR played a stronger role in mediating the influence of soil drying on AGB than LAI. Therefore, our results suggest that the association between WUE and RUE was more regulated by fAPAR than LAI, which would be important for WUE- and RUE-based models of crop production, especially under water-limited conditions.

### Source of Limitation

The two-year field experiment provided us with an opportunity to investigate the interrelationships between WUE and RUE and their underlying mechanisms across leaf and canopy scales under soil drying. Nevertheless, some limitations would still exist in the implication and generalization of the results, even though data was collected from standardized experimental measurements and after strict data quality control and analysis. Neglecting the differences in meteorological conditions between 2013 and 2014 growing seasons may be a source of uncertainties. As the three-leaf and seven-leaf stage of maize was only evaluated in a single growing season, which might not capture the effects of the year and its interaction with a development stage. Furthermore, only one maize genotype was adopted in this study, the evaluation of the cultivar variations was not conducted. Therefore, future studies can address the effects of meteorological conditions and genotypes on the interaction between WUE and RUE and their underlying mechanisms.

## Conclusion

To understand the interrelationship between WUE and RUE and their underlying mechanisms across the leaf and canopy levels under the soil drying process, a field experiment with different irrigation regimes on maize was designed and conducted during two growing seasons (2013–2014) on North China Plain. The results indicated that leaf WUE increased with irrigation reduction at an early stage, while decreased with irrigation reduction at a later stage. Leaf RUE decreased with irrigation reduction during the progressive soil drying process. Maize canopy traits (e.g., AGB, LAI, fAPAR, and *k*) were significantly decreased by water deficit regardless of the growth stage when soil drying was applied. The relationships between WUE and RUE were linear across the leaf and canopy scales under different soil drying patterns. However, the interrelationship between canopy WUE and RUE unexpectedly appeared to be positive when soil drying was applied at the three-leaf stage, while negative when soil drying was applied at the seven-leaf stage. Canopy conductance (*g*_*c*_) was more related to fAPAR than LAI. The effects of ET and IPAR on AGB were achieved *via* fAPAR more than LAI. Our finding demonstrated that the association between canopy WUE and RUE was more regulated by fAPAR than LAI under soil drying.

## Data Availability Statement

The original contributions presented in the study are included in the article/supplementary material, further inquiries can be directed to the corresponding author.

## Author Contributions

GZ, HZ, LZ, and MZ made a substantial contribution to conception and experimental design, and critically revised the manuscript. HZ, XL, and YJ performed the experiment. HZ and GZ analyzed the data and wrote the manuscript. All authors contributed to the article and approved the submitted version.

## Conflict of Interest

The authors declare that the research was conducted in the absence of any commercial or financial relationships that could be construed as a potential conflict of interest.

## Publisher’s Note

All claims expressed in this article are solely those of the authors and do not necessarily represent those of their affiliated organizations, or those of the publisher, the editors and the reviewers. Any product that may be evaluated in this article, or claim that may be made by its manufacturer, is not guaranteed or endorsed by the publisher.

## Abbreviations

ASWC: available soil water content
AGB: aboveground biomass
DAS: days after sowing
ET: evapotranspiration
fAPAR: fraction of absorbed photosynthetically active radiation
IPAR: the cumulative amount PAR intercepted by the canopy
LAI: leaf area index
NCP: North China Plain
PAR: photosynthetically active radiation
RUE: radiation use efficiency
RUE_AGB_: radiation use efficiency of AGB
RUE_leaf_: leaf radiation use efficiency
SFM: soil fresh mass
SDM: soil dry mass
WP: wilting point
FC: field capacity
SWC: soil water content
SWS: soil water storage
VPD: vapor pressure deficit
WUE: water use efficiency
WUE_AGB_: water use efficiency of AGB
WUE_leaf_: leaf water use efficiency.
